# Vitamin D and Multiple Myeloma: A Scoping Review

**DOI:** 10.3390/curroncol30030248

**Published:** 2023-03-11

**Authors:** Naghmeh Mirhosseini, Athanasios Psihogios, Meagan D. McLaren, Dugald Seely

**Affiliations:** 1 The Patterson Institute for Integrative Oncology Research, Toronto, ON M2K 1E2, Canada; 2 Kingston Integrative Healthcare Center (KIHC), Kingston, ON K7L 4T6, Canada; 3 School of Public Health, University of Saskatchewan, Saskatoon, SK S7N 2Z4, Canada; 4 The Centre for Health Innovation, Ottawa, ON K2P 0M7, Canada; 5 Ottawa Hospital Research Institute, Ottawa, ON K1Y 4E9, Canada

**Keywords:** vitamin D, multiple myeloma, oncology, hematology, scoping review

## Abstract

As the global incidence of multiple myeloma (MM) increases, the identification of modifiable risk factors for disease prevention becomes paramount. Maintaining optimal vitamin D status is a candidate for prevention efforts, based on pre-clinical evidence of a possible role in disease activity and progression. A structured scoping review was performed to identify and describe human-level research regarding the association between vitamin D and MM risk and/or prognosis. Searches of three databases (OVID-Medline, OVID-Embase, and OVID-Cochrane Library) yielded 15 included publications. Vitamin D deficiency is fairly common among patients with MM, with 42.3% of participants in the studies identified as having a vitamin D deficiency. No included publication reported on vitamin D status and the risk of developing or being newly diagnosed with MM. Possible associations with vitamin D that warrant future exploration include the incident staging of MM disease, the occurrence of peripheral neuropathy, and survival/prognosis. Vitamin D receptor (VDR) polymorphisms associated with MM also warrant further investigation. Overall, this scoping review was effective in mapping the research regarding vitamin D and MM and may help support new hypotheses to better describe this association and to better address identified knowledge gaps in the literature.

## 1. Introduction

Multiple myeloma (MM) is a hematologic malignancy, characterized by abnormal clonal plasma cells which grow uncontrollably in bone marrow, leading to destructive bone lesions, anemia, kidney disease, and hypercalcemia [1]. The global incidence of new cases is estimated to be just over 588,000 people annually [1]. MM is reported to be on the rise in developed countries, with global incidence increasing by 126% between 1990 and 2016 [2]. Non-modifiable risk factors in the development of MM include age, sex, race, and family history [2]. Modifiable risk factors include occupational hazards, chemical or pesticide exposure, obesity, and other lifestyle choices [3]. Prognostic risk factors include cytogenic abnormalities such as the presence of del(17p) or p53 mutation [4], age [5], tumour load [5], and a high plasma cell proliferation index [5]. Furthermore, it is important to consider the microenvironment as it relates to the progression and/or disease activity of MM, such as the role of microRNAs (miRNAs) which can direct the regulation of gene expression. There is a considerable body of evidence for how miRNAs may support the pathogenesis of MM through deregulation and subsequent malignant transformation [6]. Mounting evidence suggests that vitamin D may play a pivotal role in the regulation of miRNA networks which influence both physiologic homeostasis and disease processes [7].

Standard first-line (induction) therapy for MM involves a combination of a proteasome inhibitor, an immunomodulatory agent, and dexamethasone which is associated with a median progression-free survival of 41 months [1]. MM is still considered an incurable disease [8], and identification of additional modifiable factors, for both risk and prognosis, would allow for the development of novel approaches for this highly debilitating disease.

Pre-clinical research indicates that vitamin D may be meaningfully involved in the development and progression of MM. Relevant mechanisms whereby this vitamin may play such a role involve the differentiation of malignant MM cells; modulation of the immune response; and possibly through synergy with conventional treatment [9]. A more thorough synthesis of the clinical research would aid in determining if vitamin D status and/or supplementation produces clinically meaningful changes in the incidence and/or prognosis for patients with MM. To our knowledge, there is no review which specifically identifies and describes human-level research regarding the association between vitamin D and MM risk and/or prognosis. For these reasons, we conducted a structured and methodological scoping review to determine what exists in the published, peer-reviewed, literature on this topic to better describe the relevant research and facilitate meaningful hypothesis generation.

### Objective

The objective of this study is to summarize the extent, range, and nature of research activity [10,11] human-level, peer-reviewed, published research on the association between vitamin D and: (1) the risk of being diagnosed with MM and (2) prognosis and symptom burden for patients. In this review we have also described data related to vitamin D deficiency prevalence among patients with MM.

## 2. Materials and Methods

### 2.1. Methodological Approach

A structured and methodological scoping review was conducted, guided by PRISMA Extension for Scoping Review (PRISMA-ScR) procedures, to identify, describe, and map the peer-reviewed literature landscape [11] within the area of vitamin D and MM (including smoldering myeloma and MGUS (monoclonal gammopathy of undetermined significance)) risk, prevalence, and prognosis/clinical outcomes. A protocol for this scoping review was not published, and as this was not a formal systematic review, it was not registered with PROSPERO.

### 2.2. Information Sources

Three bibliographic databases (OVID-Medline, OVID-Embase, and OVID-Cochrane Library) were searched for published, peer-reviewed, literature on the 18 August 2021, without time period restrictions. The retrieved records were initially deduped using Zotero (referencing software) and then uploaded to Rayyan, a free online screening tool for conducting reviews [12].

### 2.3. Database Search Terms

Specific search terms, filters, punctuation, and conventions were applied to comprehensively explore the literature landscape. An asterisk (*) was used to broaden the search by identifying words that began with the same letters but could have different endings/suffixes. The convention “mp” (multiple places) prompted searching of multiple areas of searched records, including the title, abstract, subject heading, etc. The convention “exp” (explodes) was used to expand the search based on a specific term within the vocabulary hierarchy. Two Boolean operators (“AND” and “OR”) were used to narrow down, refine or exclude certain terms within each database. All three databases were searched using the following specific terms: “Vitamin D *”, “Calciferol *”, “cholecalciferol *”, “1,25-dihydroxycholecalciferol”, “Calcitriol *”, “vitamin D receptor *”, “VDR”, “multiple myeloma *”, “myeloma *”, “Monoclonal gammopathy of undetermined significance”, “MGUS”, “Monoclonal gammopathy of unknown significance”, “smoldering multiple myeloma”, and “SMM”. MeSH terms were then searched in the OVID-Medline and OVID-Embase databases (the OVID-Cochrane Library database did not support the use of MeSH terms), including “vitamin D”, “vitamin D deficiency”, and “multiple myeloma”. The unique MeSH term “dietary supplements” was identified and applied in OVID-Medline.

### 2.4. Selection of Sources of Evidence

Initially, retrieved record titles and abstracts were screened independently by two reviewers (NM and AP) applying the following pre-specified inclusion and exclusion criteria:

Include if:

- The primary intervention, and/or exposure under observation, and/or agent under study is vitamin D (all naturally occurring forms) and/or vitamin D receptor (VDR) related.
- The primary population under observation includes participants with multiple myeloma (MM), and/or monoclonal gammopathy of undetermined significance (MGUS), and/or smoldering myeloma (SM).
- The primary outcome includes a clinical outcome (mortality, risk of progression, QOL, symptom management, etc.) for patients with MM, MGUS, and/or SM.
- The record is a peer-reviewed study published in one of the authors’ proficient languages (English, French, or Persian).
- The record is considered primary research (experimental studies, observational studies, systematic reviews, meta-analyses, etc.) and a full-text manuscript is accessible for review.

Exclude if:

- The record is sourced from grey literature.
- The record is non-primary research (e.g., opinion pieces, conference presentations, narrative reviews, letters to the editor, etc.).
- The record is a non-peer reviewed study (e.g., pre-print).
- Synthetic vitamin D analogues are the primary focus of the study.
- The record only reports on pre-clinical data (cell-lines, in vitro, animal studies, etc.).
- The record does not report on clinical endpoints (e.g., only reporting on osteoblast activity, biomarker studies, etc.)

The full texts of the initially included studies were subsequently screened by two independent reviewers (NM and AP) for inclusion, with a third resolving any conflicts (DS). All studies that remained after screening underwent data extraction by two reviewers (NM and MDM), with a third performing spot verification (AP).

### 2.5. Data Charting Process and Items

Information from each included study was collected and inputted into a data extraction form with prespecified categories. Data extraction endpoints included reference details (author, publication year, region, and study design), study population and sample size, intervention details for experimental studies (type of vitamin D supplement, dose, duration of treatment, and control or placebo details), serum 25(OH)D status, changes in serum 25(OH)D, clinical outcomes (mortality, progression, QOL, other anti-cancer effects, changes to bone health, and other health status changes), and safety outcomes/toxicity.

### 2.6. Synthesis and Presentation of Results

All of the included publications were reviewed, and relevant characteristics were collected and presented, including country, study design, data collection period, and study population (number of participants, sex, and age distribution as reported in the retrieved record) (Table 1). After identifying which country each study was conducted in, the investigators confirmed and reported if the region was a member of the Organisation for Economic Co-Operation and Development (OECD) [13] to better conceptualize the socioeconomic status characteristics of the study population (Table 1). Studies were grouped based on their definition of vitamin D sufficiency and deficiency (described values are reported verbatim as they were in the original publications) (Table 2). Studies were grouped and presented based on reported outcomes, including: (1) those reporting on the prevalence of vitamin D deficiency among patients (Table 3); (2) those reporting on the association between vitamin D status and the risk of developing MM; and (3) those reporting on the association between vitamin D status and prognosis and/or clinical outcomes (Table 4). Implications of VDR polymorphisms are summarized for consideration based on the publications that explored genetic differences. Descriptive statistics were used to summarize trends where applicable and data were presented in detail in tabular format and discussed narratively through text.

## 3. Results

### 3.1. Selection of Sources of Evidence and Included Studies

**Figure 1:**
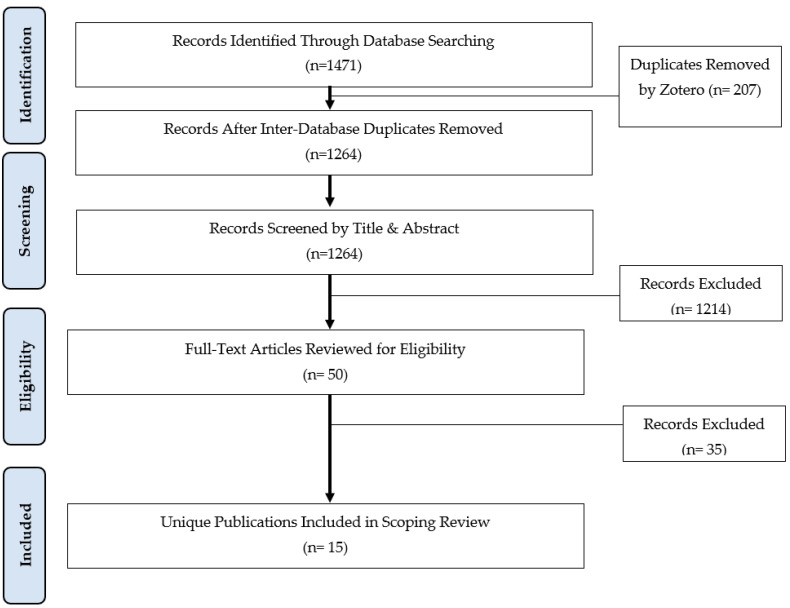
PRISMA flowchart.

The applied search strategy, after intra-database deduplication, yielded 322 records from OVID-Medline, 1083 records from OVID-Embase, and 66 records from OVID-Cochrane Library, producing a total of 1471 studies. Records from all three deduped databases were then amalgamated in Zotero and underwent inter-database deduplication, removing 207 studies, leaving 1264 which were subsequently uploaded to Rayyan for screening. Abstract and title screening (blinded) performed independently by two investigators (NM and AP) excluded 1214 records, leaving 50 to screen by full text, of which 35 were subsequently excluded, yielding 15 publications [14,15,16,17,18,19,20,21,22,23,24,25,26,27,28] for this scoping review. One study produced two publications reporting on different outcomes [21,22]; hence 14 separate studies were identified yielding 15 overall publications. A detailed description of the screening process is presented via a PRISMA flow-chart (Figure 1). The study characteristics of the 15 included publications are described in Table 1.

### 3.2. Characteristics of the Included Studies

All of the identified studies in this scoping review applied an observational study design, including four cross-sectional studies (yielding five publications) [19,21,22,24,28], three retrospective cohorts [14,17,27], two case-control studies [16,25], one cross-sectional study with an additional retrospective chart review [18], one retrospective chart review [26], one matched controlled cohort [23], one prospective cohort [20], and one case report [15]. No experimental studies were identified in the literature landscape. This represents an identified knowledge gap regarding study design in the research. The region producing the most publications (n = 3) on the topic of vitamin D and MM is the United States of America (USA) [14,19,26], with the majority of studies (n = 10) conducted in regions that are currently members of the OECD [14,15,17,18,19,20,24,26,27,28]. Eleven publications (ten conducted studies) reported on the prevalence of vitamin D deficiency (as a tally) among participants (Table 3) [14,17,18,19,20,21,22,24,26,27,28], none reported on the risk of developing MM and vitamin D status, and eleven publications (ten conducted studies) reported on prognosis and/or clinical outcomes associated with vitamin D status [14,15,17,19,20,21,22,24,26,27,28]. Three publications explored the implications of VDR differences rather than vitamin D status in patients with MM [16,23,25]. A summary of included study characteristics is presented in Table 1.

### 3.3. Classification of Study Participants Based on Vitamin D Status

The definition of vitamin D status as either “sufficient” or “deficient” varied between publications according to both cutoff values and units of measurement. The most common paired cutoff values within the same publication (n = 4) [18,21,22,27] were <20 ng/mL 25(OH)D (deficient) and >30 ng/mL 25(OH)D (sufficient). After conversion of ng/mL to nmol/L (and vice versa), the most common individual cutoff value for vitamin D deficiency from all publications was <50 nmol/L (<20 ng/mL) 25(OH)D (n = 9) [14,18,19,21,22,24,26,27,28] and >75 nmol/L (>30 ng/mL) 25(OH)D for vitamin D sufficiency (n = 6) [17,18,21,22,27,28]. The three publications that reported on VDR status did not assess vitamin D status against prespecified values [16,23,25]. Vitamin D definitions, as reported and discussed in the included publications, are presented in Table 2. No standard vitamin D status definition was discovered in the literature landscape evidenced by the heterogeneity between the included publications.

### 3.4. Prevalence of Vitamin D Deficiency

Ten publications reported on the prevalence (as a tally) of vitamin D deficiency within their sample, applying the status definitions described in Table 2 [14,17,19,20,21,22,24,26,27,28]. A detailed report, including tallies and percentages, of vitamin D deficiency prevalence reported in each publication is described in Table 3. Note that the case report by Clement et al. [15] is not included in Table 3 as it only reports on a single individual who was found to be vitamin D deficient (<20 nmol/L) and the three publications [16,23,25] exploring VDR activity did not report on deficiency prevalence and were thus also omitted. One publication which explored the prevalence of vitamin D deficiency in patients with MM and bone metastasis did not report prevalence as a tally but rather as a mean (mean 25-OH-D = 14.8 ng/mL (±6.3 ng/mL), and hence, they were not included in Table 3 [18]. Based on eligible publications reporting a tally prevalence presented in Table 3, where percentages could be calculated, the median percentage of patients with vitamin D deficiency was 42.3% (range: 23.7% [14] to 100% [21,22]). Nine publications (the majority) reported that 25% or more of patients were observed to be vitamin D deficient [17,19,20,21,22,24,26,27,28]. Sex differences were observed, with the median percentage of participants classified as vitamin D deficient among females being 44.95% (range: 34.3% [14] to 54.6% [24]) and 55.1% (range: 45.4% [24] to 65.7% [14]) among males.

### 3.5. Association between Vitamin D Status and the Risk of Developing MM

None of the identified records explored or reported on the association between vitamin D status and the risk of developing/being newly diagnosed with MM. While some publications examined the association between vitamin D status and different characteristics of disease, such as stage (presented in the subsequent section and Table 4), the risk of MM was absent from the mapped literature landscape in this scoping review. This represents an identified knowledge gap in the research.

### 3.6. Associations between Vitamin D Deficiency and MM Prognosis, Clinical Outcomes, and Disease Sequelae

A detailed description of different outcomes reported in each study is described in Table 4. Three studies reported on the association between vitamin D levels and survival [17,20,26], with one reporting no significant association [17], though the sample size was small and likely underpowered and two reported an association between deficiency and poorer survival endpoints [20,26]. Two studies (three publications) [21,22,24] reported no association between vitamin D status and MM stage, while three reported higher stages observed with lower levels [14,23,27]. Only one study reported a significant association between vitamin D and bony disease (fractures and lytic lesions) [27], while four studies (five publications) reported no significant association [14,17,21,22,24]. Observations from studies exploring peripheral neuropathy were conflicting, with one reporting an association [19] with severity while the other did not [24], and two reporting deficiency increasing occurrence [24,28] while one found no association [19]. Two [16,25] of the three publications exploring VDR activity that did not specifically report on vitamin D status are only discussed in the subsequent section. Maier et al. [18] only reported on vitamin D deficiency prevalence (reported on in Table 3) and thus their study not included in Table 4.

### 3.7. Vitamin D Receptors and MM

Three publications explored the role of VDR activity and genetic profile differences (e.g., polymorphisms) in patients with MM [16,23,25].

One study explored VDR gene polymorphisms in 40 patients with MM and 83 matched healthy controls [25]. The authors report observing a significantly higher frequency in the MM group compared to controls of the A allele at the BsmI site (8.7% compared to 2.4%) and the C allele at the TaqI site (10.5% compared to 3.6%). These two identified alleles with a higher frequency in MM patients were found to be significantly associated with an increased risk of disease (*p* = 0.025 and *p* = 0.030, respectively).

A similarly designed study included 75 patients with MM and 150 controls from the general population to explore three VDR polymorphisms (ApaI, BsmI, and FokI) [16]. The following genotypic VDR distributions were not significantly associated with MM, ApaI AA/Aa/aa genotypes, and BsmI BB/Bb/bb genotypes (*p* > 0.05). ApaI a allele and BsmI b allele were significantly associated with MM (OR: 1.53, *p* = 0.03 and OR: 1.71, *p* = 0.007, respectively). FokI polymorphisms were found to be significantly associated with MM (*p* = 0.000032), with the ff genotype compared to the FF genotype significantly associated with an increased risk (OR: 5.33, *p* < 0.0001).

The final VDR study that included 75 newly diagnosed MM patients and 75 matched controls reported that the single nucleotide polymorphisms Ff and ff, Aa and aa, and Bb and bb genotypes are significantly associated with an increased risk of disease (*p* < 0.05). Furthermore, the alleles FokI f, ApaI a, and BsmI b were found to be significantly associated with MM occurrence (*p* < 0.05).

## 4. Discussion

The research landscape mapped in this scoping review indicates that vitamin D deficiency is fairly prevalent among patients with MM, and that an individual’s sufficiency status may be associated with ISS stage at diagnosis, the occurrence of peripheral neuropathy, and with less certainty and reduced survival/prognosis. Based on the identified studies, vitamin D status does not appear to be significantly associated with bony disease such as lytic lesions or fractures. Based on the relatively few studies exploring VDR differences among patients with MM, when compared to healthy controls, the alleles FokI f, ApaI a, and BsmI b were consistently associated with an increased risk of MM. The literature exploring and describing the risk of developing MM based on vitamin D status was absent from the described landscape. A standard definition for vitamin D status (deficient vs. sufficient) across studies was not observed.

Vitamin D has been observed in pre-clinical settings to influence important disease components of MM, such as the differentiation of malignant cells and immunomodulation [9]. In real world settings, the interaction between vitamin D and the individual may have the potential to translate into clinically meaningful changes to their disease trajectory. For example, MM survival predictions are partially dependent on stage, with ISS stage I associated with an overall 5-year survival rate of 82% compared to 40% for ISS stage III [2]. This scoping review identified evidence of an association between vitamin D status and ISS staging at diagnosis, with lower levels often significantly associated with higher MM stage. As part of ongoing public health initiatives to maintain population vitamin D levels at adequate levels to reduce disease rates and burden [29], MM may be an important additional target for prevention efforts.

Beyond survival, as patients with MM are living longer with an incurable hematological disease, increased symptom and disease burden is often reported which notably can deteriorate quality of life (QOL) [30]. Observed in up to 54% of patients with MM, peripheral neuropathy due to disease activity and/or treatment is a common issue that has the potential to cause pain and debilitation [31]. In the described literature landscape, some evidence of a protective effect of vitamin D sufficiency was found for the occurrence of peripheral neuropathy in patients with MM. Interestingly, the majority of evidence did not show an association between vitamin D and bony disease, even though it is well established to have a role in bone health [32]. This may be in part due to the complex interaction between vitamin D and other essential nutrients to maintain bone health, as evidenced by the absence of benefits when it supplemented in isolation to reduce fractures, but when calcium is added, beneficial effects are observed in the general population [33]. Of note, in the one case report identified in this scoping review, an individual with MM who supplemented with vitamin D did report reduced musculoskeletal pain [15].

As with other diseases, intervention responses are often influenced by genetic variability within the population [34] and may possibly influence the observed effects of vitamin D for patients with MM. VDR polymorphisms have been reported to be associated with altered disease risk, including breast cancer [35], non-melanoma skin cancer [36], and an array of tobacco-related cancers [37]. The literature identified in this scoping review regarding VDR polymorphisms and MM risk were fairly consistent across the studies, indicating a possible new genetic target for future risk stratification and the identification of best responders to vitamin D supplementation. Based on the findings of this scoping review, it may be beneficial to consider genomic directed stratification in the design of future clinical trials in order to determine the extent of influence that VDR polymorphisms exert on MM risk and/or management.

Although successful in mapping the literature landscape on this topic, the following limitations are considered. The internally generated protocol used to conduct this scoping review was not registered with PROSPERO. Furthermore, this scoping review did not conduct a formal environmental scan, only including peer reviewed published articles and not grey literature which may have omitted relevant information. It is important to note that scoping reviews are generally considered to be hypothesis-generating, not hypothesis-testing, and while the literature landscape was mapped, no conclusive comments can be made [38]. No human-level studies reported on the association between vitamin D deficiency and the risk of developing MM, therefore no comment could be made one of our sub-objectives (disease risk).

Review of the present literature landscape was effective in identifying literature exploring the association between vitamin D status (and to a lesser degree, VDR polymorphisms) and MM. The results of this scoping review can be used to form new hypotheses related to vitamin D and MM, with emphasis placed on those with the most consistent evidence (stage of disease, peripheral neuropathy, and survival), focusing on experimental studies which are presently absent from the described landscape.

One possible vein of future investigation could include the application of novel miRNA-based nano-carrier strategies to deliver vitamin D to specific targets within the tumour microenvironment in order to influence gene expression [7]. This is not something we explored in this scoping review; however, the strategy warrants consideration in the application of further preclinical and clinical research.

## 5. Conclusions

In addition to the already identified risk factors that have been identified to substantially impact risk and prognosis, further work is warranted to assess the potential impact of vitamin D as a low-cost intervention in the setting of reducing MM risk as well as in impacting the progression and morbidity of this disease. Determining optimal dosing and vitamin D status amongst patients at risk and those with a multiple myeloma diagnosis may provide promising additional insights to address this type of cancer.

## Figures and Tables

**Table 1:** Characteristics of the included publications.

Reference	Country	Study Design	Vitamin D Collection Period	MM Participant Characteristics
Ng et al. (2009) [14]	United States of America (OECD Member)	Observational; retrospective cohort	Serum 25-hydroxyvitamin D levels measured from 1 January 2004 to 31 December 2008 (4 years)	N = 148 patients with MM (newly diagnosed) Females (n = 56) Males (n = 92) Age range: 56.6 to 64.7 years of age ^‡^
Clement et al. (2011) [15]	Australia (OECD Member)	Case report	Collection of data occurred in June 2012	63-year-old male with MM receiving Bortezomib
Shafia et al. (2013) [16]	India (Not an OECD Member)	Observational; case-control	Vitamin D levels not measured	N = 75 patients with MM Females (n = 28) Males (n = 47) Mean age: 54 years of age
Lauter and Schmidt-Wolf (2015) [17]	Germany (OECD Member)	Observational; retrospective cohort	Serum 25-hydroxyvitamin D levels measured from December 2007 to December 2014 (7 years)	N = 83 patients with MM Females (n = 32) Males (n = 51) Mean age: 66.3 years of age (range: 43–86)
Maier et al. (2015) [18]	Germany (OECD Member)	Observational: cross-sectional (additional retrospective chart review also conducted)	Serum 25-hydroxyvitamin D levels measured from 1 January 2011 to 31 December 2012 (1 year)	N = 49 patients with MM experiencing bone metastases Females (n = 109) Males (n = 87) Mean age: 58 years of age (±8.1 years)
Wang et al. (2016) [19]	United States of America (OECD Member)	Observational; cross-sectional	Not reported	N = 109 patients with MM Females (n = 40) Males (n = 59) Mean age: 66 years of age (range: 42–89)
Eicher et al. (2020) [20]	Switzerland (OECD Member)	Observational; prospective cohort	Not reported	N = 104 patients with MM Females: n/a * Males: n/a * Age range: n/a *
Graklanov and Popov (2020) (Publication #1 [21] and #2 [22])	Bulgaria (Not an OECD Member)	Observational; cross-sectional	Serum 25-hydroxyvitamin D levels measured from November 2014 to April 2016 (29 months)	N = 37 patients with MM Female (n = 19) Male (n = 18) Median age: 68 years of age (range: 38–86) ^‡^
Kumar et al. (2020) [23]	India (Not an OECD Member)	Observational; matched controlled cohort	Vitamin D levels not measured	N = 75 patients with MM Female (n = 21) Male (n = 54) Mean age: 57 years of age (range: 38–78)
Nath et al. (2020) [24]	Australia (OECD Member)	Observational; cross-sectional	Serum 25-hydroxyvitamin D levels measured from March to July 2018 (5 months)	N = 41 patients with MM Female (n = 9) Male (n = 21) Age range of study population: 45–90 ^‡^
Rui et al. (2020) [25]	China (Not an OECD Member)	Observational; case-control	Serum 25-hydroxyvitamin D levels measured from August 2014 to February 2016 (18 months)	N = 40 patients with MM Females (n = 17) Males (n = 23) Mean age: 59.5 years of age (range: 34–81)
Yellapragada et al. (2020) [26]	United States of America (OECD Member)	Observational; retrospective chart review	Not reported	N = 1889 patients with MM Females (n = 60) Males (n = 1829) Mean age at diagnosis: 68.9 years of age (±10.2 years)
Sincan and Erdem (2021) [27]	Turkey (OECD Member)	Observational; retrospective cohort	Not reported	N = 184 patients with MM Female (n = 55) Male (n = 129) Mean age: 68.7 years of age (±10.47 years)
Oortgiesen et al. (2022) [28]	Netherlands (OECD Member)	Observational; cross-sectional	Serum 25-hydroxyvitamin D levels measured from January 2017 to August 2018 (20 months)	N = 120 patients (105 with MM and 15 with smoldering myeloma) Female (n = 51) Male (n = 69) Mean age: 68 years of age (±7.7 years)

MM: multiple myeloma; OECD: Organisation for Economic Co-Operation and Development. * study included non-MM participants and tally not available as values were not separated out and reported specifically for patients only with MM. ^‡^ Mean age not reported.

**Table 2:** Definitions of vitamin D status.

Reference	Definition of Vitamin D Deficiency and Sufficiency
Ng et al. (2009) [14]	Deficient: <50 nmol/L 25(OH)D Sufficient: ≥50 nmol/L 25(OH)D
Lauter and Schmidt-Wolf (2015) [17]	Deficient: <10 ng/mL 25(OH)D Sufficient: >30 ng/mL 25(OH)D
Oortgiesen et al. (2022) [28]	Deficient: <50 nmol/L 25(OH)D Sufficient: >75 nmol/L 25(OH)D
Nath et al. (2020) [24]	Deficient: <50 nmol/L 25(OH)D Sufficient: No formal definition reported
Maier et al. (2015) [18]	Deficient: <20 ng/mL 25(OH)D Sufficient: >30 ng/mL 25(OH)D
Sincan and Ederm (2021) [27]	
Graklanov and Popov (2020) [21,22]	
Wang et al. (2016) [19]	Deficient: <20.0 ng/mL 25(OH)D Sufficient: No formal definition reported
Yellapragrada et al. (2020) [26]	
Eicher et al. (2020) [20]	Divided groups into “normal” and “low”: Normal: >52 nmol/L 25(OH)D Low: ≤52 nmol/L 25(OH)D
Clement et al. (2011) [15]	Deficient: <36 nmol/L 25(OH)D Sufficient: No formal definition reported
Shafia et al. (2013) [16]	Not applicable—Vitamin D receptor study
Kumar et al. (2020) [23]	
Rui et al. (2020) [25]	

**Table 3:** Prevalence of vitamin D deficiency among MM study participants.

Reference	Prevalence of Vitamin D Deficiency N/Total (%)
Ng et al. (2009) [14]	35/148 (23.7%) Female: 12/35 (34.3%) Male: 23/35 (65.7%)
Lauter and Schmidt-Wolf (2015) [17]	27/83 (32.5%) Female: 13/27 (48.2%) Male: 14/27 (51.8%)
Wang et al. (2016) [19]	47/111 (42.3%) * Female: Not reported Male: Not reported
Eicher et al. (2020) [20]	81/183 (44.3%) Female: 31/81 (38.3%) Male: 50/81 (61.7%)
Graklanov and Popov (2020) [21,22]	37/37 (100%) * Female: 19/37 (51.4%) Male: 18/37 (48.6%)
Nath et al. (2020) [24]	11/41 (26.8%) Female: 6/11 (54.6%) Male: 5/11 (45.4%)
Yellapragada et al. (2020) [26]	583/1889 (30.9%) Female: Not reported Male: Not reported
Sincan and Erdem (2021) [27]	148/184 (80.4%) * Female: Not reported Male: Not reported
Oortgiesen et al. (2022) [28]	60/120 (50.0%) * Female: 25/60 (41.7%) Male: 35/60 (58.3%)

* Those reported to be “seriously deficient (or just deficient)” (<25 nmol/L) and “deficient (or insufficient)” (25–50 nmol/L) grouped together.

**Table 4:** Vitamin D deficiency (VDD) and MM prognosis, clinical outcomes, and disease sequelae.

Reference	Association with Survival	Association with Staging	Association with Relevant Hematological Markers	Association with Clinical Outcomes/Symptoms
Ng et al. (2009) [14]	N/A	VDD associated with higher ISS staging (*p* = 0.03); Unadjusted OR for being VDD was 3.56 for ISS stage III compared to stage I	VDD associated with higher mean CRP level (*p* = 0.02) and lower albumin levels (*p* = 0.003)	No association found for VDD and skeletal morbidity (fractures and lytic lesions)
Clement et al. (2011) [15]	N/A	N/A	N/A	Four months of supplementation with vitamin D (3000 IU) resulted in one individual (case report) reporting reduced generalized musculoskeletal pain
Lauter and Schmidt-Wolf (2015) [17]	Of seven participants who died of progressive disease, five were vitamin D insufficient, one was deficient, and one was sufficient (*p* = 0.932)	N/A	MM patients with VDD had higher plasma cells in bone marrow (44.8%) compared to those classified as sufficient (13.3%)	No association found for vitamin D status and lytic bone lesions or disease activity. MM patients found to be without renal insufficiency were also found to have significant increases in vitamin D levels after supplementation
Maier et al. (2015) [18]	N/A	N/A	N/A	Among MM patients with bone lesions, the mean vitamin D level was reported to be deficient (14.8 ng/mL)
Wang et al. (2016) [19]	N/A	N/A	N/A	No association found between VDD and the occurrence of either motor PN or sensory PN. VDD was associated with the severity of PN (>grade 2) for both motor PN (*p* = 0.042) and sensory PN (*p* = 0.009). No association was observed for pain
Eicher et al. (2020) [20]	MM patients who underwent chemotherapy treatment and ASCT who had VDD had significantly shorter PFS (median 16.0 months) compared to those with normal levels (median 19.5 months) (*p* = 0.0412) OS was shorter among VDD patients (20.4 months) compared to those with normal levels (21.4 months) (*p* = 0.049)	N/A	N/A	N/A
Graklanov and Popov (2020) (Publication 1 [21] and 2 [22])		No association found between vitamin D status and ISS stage	No association found between vitamin D status and hemoglobin levels	No association found between vitamin D levels and response to treatment. No association found between vitamin D levels and bone disease
Kumar et al. (2020) [23]	N/A	VDD associated with higher stage of disease (*p* < 0.001)	N/A	N/A
Nath et al. (2020) [24]		No association found between vitamin D status and ISS stage	No association between vitamin D status and creatinine or albumin	Lower vitamin D levels were significantly associated with lower performance status (ECOG ≥ 2) (*p* = 0.003). The rate of peripheral neuropathy was significantly higher among patients with VDD (73%) compared to those who were not deficient (33%) (*p* = 0.03). A non-significant association was noted for VDD and self-reported peripheral neuropathy symptom severity (*p* = 0.08). No significant association between VDD and skeletal morbidity or 5-year fracture history was observed
Yellapragada et al. (2020) [26]	VDD was significantly associated with worse OS (median 3.10 years, 95% CI: 2.73 to 3.52) compared to patients with normal levels (median 3.91 years, 95% CI: 3.59 to 4.38) (*p* = 0.002). The estimated mortality risk for VDD was a 24% increase (HR: 1.24; *p* = 0.02). Log-transformed vitamin D level was reported to be a significant predictor of survival in White patients in univariate (HR: 0.77, *p* = 0.002) and multi-variate (HR: 0.74, *p* = 0.009) analysis, but not in African American patients for either analysis	N/A	N/A	N/A
Sincan and Erdem (2021) [27]		Lower levels of vitamin D were associated with increased ISS stage (*p* = 0.01)	No association was observed between vitamin D levels and hemoglobin, albumin, or creatinine. Lower levels of vitamin D were significantly associated with a higher mean % of plasma cells in bone marrow (*p* = 0.02)	Lower vitamin D levels were significantly associated with bone fracture (*p* = 0.007) and the presence of lytic bone lesions (*p* = 0.01)
Oortgiesen et al. (2022) [28]	N/A	N/A	N/A	Vitamin D levels were significantly and inversely associated with the occurrence of PN (*p* = 0.035)

VDD: Vitamin D deficiency (as defined but for each individual study), CRP: C-reactive protein, ISS: International Staging System, OR: odds ratio, N/A: not applicable, PN: peripheral neuropathy, ASCT: autologous stem cell transplantation, PFS: progression free survival, OS: overall survival, ECOG: Eastern Cooperative Oncology Group, CI: confidence interval, and HR: hazard ratio.
